# Reduced predation risk for melanistic pygmy grasshoppers in post-fire environments

**DOI:** 10.1002/ece3.338

**Published:** 2012-08-01

**Authors:** Einat Karpestam, Sami Merilaita, Anders Forsman

**Affiliations:** 1 Ecology and Evolution in Microbial Model Systems, EEMiS, School of Natural Sciences, Linnaeus UniversityKalmar, Sweden; 2 Environmental and Marine Biology, Åbo Akademi UniversityTurku, Finland

**Keywords:** Adaptation, camouflage, crypsis, evolution, fire melanism, masquerade, predation

## Abstract

The existence of melanistic (black) color forms in many species represents interesting model systems that have played important roles for our understanding of selective processes, evolution of adaptations, and the maintenance of variation. A recent study reported on rapid evolutionary shifts in frequencies of the melanistic forms in replicated populations of *Tetrix subulata* pygmy grasshoppers; the incidence of the melanistic form was higher in recently burned areas with backgrounds blackened by fire than in nonburned areas, and it declined over time in postfire environments. Here, we tested the hypothesis that the frequency shifts of the black color variant were driven, at least in part, by changes in the selective regime imposed by visual predators. To study detectability of the melanistic form, we presented human “predators” with images of black grasshoppers and samples of the natural habitat on computer screens. We demonstrate that the protective value of black coloration differs between burnt and nonburnt environments and gradually increases in habitats that have been more blackened by fire. These findings support the notion that a black color pattern provides improved protection from visually oriented predators against blackened backgrounds and implicate camouflage and predation as important drivers of fire melanism in pygmy grasshoppers.

## Introduction

Cryptic coloration provides one way in which a prey can avoid being detected and eaten by visual predators. Background matching, in which an animal possesses similar colors or patterns as its environment, is a widespread form of cryptic coloration (e.g., Kettlewell 1956; Endler 1980; Merilaita et al. 1999; Merilaita and Stevens 2011). In addition, individuals may avoid predation by not being identified as potential food items, a phenomenon known as masquerade, whereby potential prey items may resemble inedible objects found in their surroundings (e.g., Stevens and Merilaita 2009; Skelhorn et al. 2010b).

Differential predation and selection for crypsis has been invoked as an important driving force behind the spatiotemporal variation in the incidence individuals with a melanistic color form (i.e., a general or partial darkening of the color or patterning) that has been reported in local populations for many species of animals (Majerus 1998). Perhaps the best known example of adaptive temporal evolutionary shifts in melanism frequencies in response to selection for protective coloration in dark environments is the case of industrial melanism, the Peppered Moth *Biston betularia* (Kettlewell 1955, 1956; Majerus 1998; Cook et al. 2012). Similarly, it has been shown in squirrels that frequencies of melanistic individuals are positively correlated across populations with increased occurrence of wildfires (e.g., Guthrie 1967; Kiltie 1989). Furthermore, the pocket mouse *Chaeotodipus intermedius,* which has a light-colored pelage throughout most of its range where rocks are light colored, exhibits high frequencies of darker phenotypes in areas characterized by dark basalt rock (Hoekstra et al. 2004). In an experiment with plasticine models of mice, Vignieri et al. (2010) have shown that cryptic models that matched their visual background (i.e., dark models on dark soil, light models on light soil) were attacked at a lower rate than mismatching models. However, sample size in this study was fairly small.

Recently, Forsman et al. (2011) demonstrated that the frequency of melanistic morph of the pygmy grasshoppers *Tetrix subulata* is much higher (ca 50%) in populations that inhabit areas that were recently damaged by fire, than in populations that inhabit nonburnt environments (ca 9%). Furthermore, within 4 years from the fire, the frequency of melanistic individuals drops from about 50% to about 30%. Forsman et al. (2011) proposed that these microevolutionary shifts in melanism frequencies are driven in part by selection imposed by visually oriented predators, and that black coloration offered superior protection in environments where the substrate had been blackened by fire.

There is some evidence indicating differential predation on color pattern in pygmy grasshoppers. Using a combination of experimental manipulation of color patterns and capture-mark-recapture data Forsman and Appelqvist (1999) showed that color pattern differently influences survival of free ranging male and female pygmy grasshoppers in the wild. In another study, Forsman and Appelqvist (1998) exposed color manipulated pygmy grasshoppers (black or striped) to predation by domestic chicks under controlled conditions in the laboratory, and demonstrated that predation by visually oriented predators may impose correlational selection that favors certain combinations of color pattern and behavior, at the expense of other combinations. Similarly, Civantos et al. (2004) performed staged encounters between *T. subulata* and *Psammodromus algirus* lizards, and also arrived at the conclusion that the relative protective value of a given color pattern depends on the behavior of the prey animal. In a more recent study, Tsurui et al. (2010) used another species of pygmy grasshopper, *Tetrix japonica* as prey and compared the detectability of four color morphs when presented on two different visual backgrounds (grass and sand), using humans as “predators”. They found that the relative crypsis of the different color morphs was reversed between the two backgrounds. Although these studies implicate differential predation on color pattern in pygmy grasshoppers, there is as yet no firm evidence to support the notion (Forsman et al. 2011) that melanistic coloration offers better protection against visually oriented predators in postfire habitats.

In this study, we present human “predators” with images of black grasshoppers and samples of the natural habitat on computer screens to compare probability of detection and survival of black grasshoppers on black (burnt) versus greenish (nonburnt) backgrounds and investigate how the probability of detection of the black morph changes with a gradual change as the environment recovers from fire.

## Material and Methods

### Study species

*Tetrix subulata* is a widely distributed species of pygmy grasshopper (up to 15 mm long), which often inhabits damp places and lives on the soil's surface feeding on algae, short grass, moss, and partly decomposed plant and animal matter (Rehn and Grant 1955; Holst 1986). In addition, *T. subulata* exhibits color polymorphism, and a large number of discrete color morphs are present within populations. Ground color ranges from black, via various shades of brown to light gray, and almost white. Some morphs are monochrome, while others have patterning consisting, for example of a longitudinal stripe along the median pronotum, or of specks or spots of variable colors and widths on pronotum, or on the femora of the jumping legs (Nabours 1929; Karlsson et al. 2008; Caesar et al. 2010). Color morphs are under genetic influence, and morph frequencies differ both among populations and over time within populations (Karlsson et al. 2008; Forsman et al. 2011).

### Photographic sampling of the habitats

**Figure 1:**
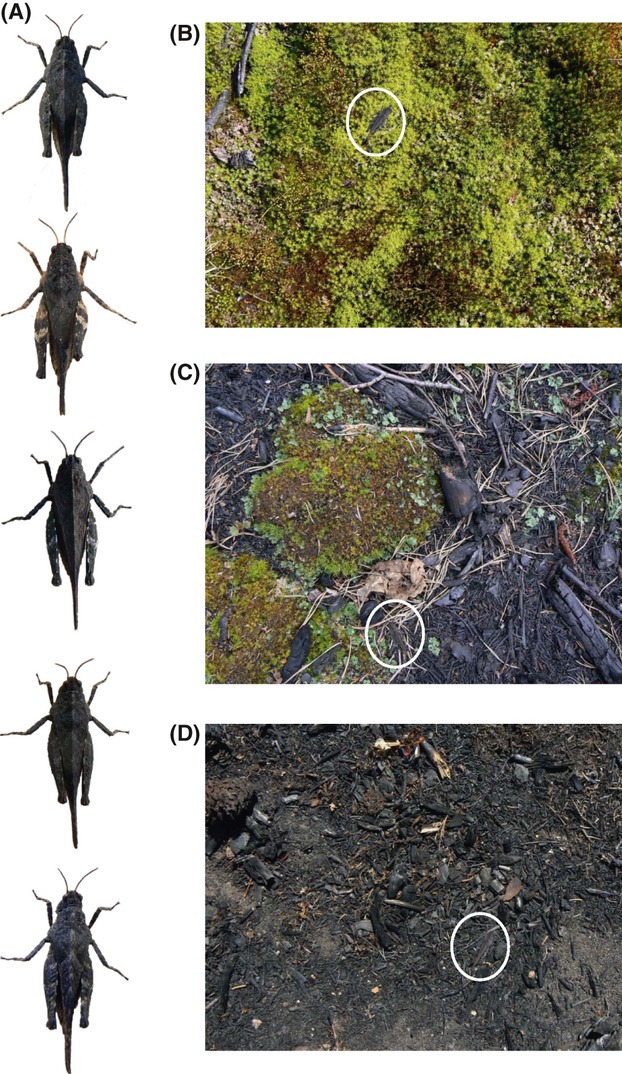
Melanistic *Tetrix subulata* pygmy grasshoppers presented on samples of their natural background. (A) Examples of individual variation among melanistic individuals. (B–E) *T. subulata* presented on images of samples of backgrounds from nonburnt environment (B), and from different successional stages in postfire environments representing (C) 50% burnt substrate and (D) 100% burnt substrate. White circles denote the location of the grasshoppers.

Photographs of the habitats were taken in summer 2010 with a digital compact camera (Panasonic Lumix DMC-TZ7, 35mm focal length; Panasonic, Osaka, Japan) using “macro” mode at a vertical distance of approximately 30 cm straight from above. The pictures were taken at three previously burnt locations in southeast Sweden (Smedjevik [N56°59.335, E16°5.743, burnt 2010], Åsjön [N56°58.685, E16°4.6213, burnt 2010] and Påryd [N56°34.5115, E15°55.547, burnt 2009]). All locations included burnt pine and spruce forests at different stages of recovery, several types of mosses, burnt and sooty material such as branches, twigs, and needles, and small streams or temporary water bodies. All localities were also surrounded by healthy forest that had not burnt recently. In all locations, pictures (300 in total) were taken randomly (while walking across the burnt area), but with the purpose of including a mixture of burnt and nonburnt areas in some pictures, and totally burnt or nonburnt areas in others. These pictures were then sorted into one of five categories of burnt backgrounds, representing different successional stages after fire events (Fig. 1).

### Collection and photography of grasshoppers

Grasshoppers were observed in all of the locations and adult, black individuals, all with a long pronotum in order to reduce variation due to shape and size, were taken to the laboratory where they were kept in rearing buckets before they were photographed (*N* = 7). Photographs were taken out of doors under natural shaded light conditions to avoid strong shading and reflections caused by direct sun light. We used live individuals to avoid unnatural body position; therefore, prior to photography sessions the grasshoppers were kept at 5°C for approximately 20 min to limit their movement ability before they were placed on a brownish piece of cardboard. After approximately 30–60 sec, grasshoppers had warmed up and started to jump again. Photography was therefore done fast, and individuals were returned to their buckets immediately after photographing. All grasshoppers were photographed in summer 2010 with the same camera as the backgrounds using “macro” mode approximately 7 cm from above.

### Image processing

Image processing was done using Adobe Photoshop CS4 (Adobe systems Incorporated, San Jose, California). Grasshopper images were cut from the backgrounds and saved on transparent background in order to later be implanted on the habitat pictures. Grasshoppers were measured for pronotum length and scaled so that they all had similar “actual sizes” in full picture view (16 ± 0.3 cm). When presented to human “predators” the size of grasshoppers was rescaled, as explained below. This was done in order to avoid bias toward easier detection of larger individuals. When necessary, background pictures were reduced or elevated brightness levels so that they all had similar brightness comparable to that of the grasshopper images.

### Programming and detection task

To test the hypothesis that black grasshoppers are harder to detect on backgrounds that were blackened by fire, we choose five different background levels. The levels were 0%, 25%, 50%, 75%, and 100% of total burnt area in the picture, and intended to represent a process of successional recovery after a fire. Thus, level 0% corresponds to nonburnt and contained different type of mosses and had a general greenness appearance, whereas level 100% contained only burnt soil and sooty material and was altogether black. Levels 25%, 50%, and 75% were intermediate and consisted of a mixture of finer or larger patches of burnt material and live vegetation, such as green mosses. The amount of black cover was estimated by eye by E. K and A. F. Seven background pictures for each of the five background levels were used for the program in the experiment (see below).

In the experiment, we used images of seven different grasshopper individuals, which represented slightly different versions of the black *T. subulata* color morph. All grasshoppers were black on the pronotum, but differed from each other in details of the patterning on the femurs of jumping legs and in general “darkness”. For example, some individuals were completely black, whereas others had white or yellow spots on the legs. We used images of several different individuals in order to increase generality of our results, and to simulate the situation in wild populations, in which there is subtle variation also within the different morph categories (Fig. 1A).

Each human test subject was randomly assigned to one of the five background levels. Each subject was presented with 15 habitat photographs, into each of which one of the seven black grasshoppers was implanted (Fig. 1B–D). The combinations of background images and grasshopper images as well as the position and rotation angle (1–360°) of the grasshopper in the background picture were randomly selected by the purpose-written program (see below). Grasshoppers were scaled to natural size and where approximately 15 mm when measured on the presentation screen. Test subjects were asked to search for the grasshopper and use the mouse to point and click it. After a click, the presentation program recorded the result (“correct” if on the grasshopper, else “wrong”) and time to detection, after which it presented a new combination of background and grasshopper. If a test subject did not click the mouse within 60 sec from the beginning of a presentation, the presentation program recorded the result of the trial as “wrong” and detection time as 60 sec, and a new combination of images was presented. In the survival analysis described below, we combined the data for time to detection and correctness of the response.

Prior to the experiment, each test subject was given a short practice block which started with verbal explanation of the task, and general instructions, in a darkened room in order to adapt the subjects to the light conditions (dark to avoid reflections on the screen) in the room. The second part included familiarization with the grasshoppers, in which a grasshopper image was first presented on a plain white background on otherwise gray picture for 20 sec, in the actual size they appeared in the experimental trials. This was followed by two training presentations on the images of real backgrounds, one on level 0% burnt and the second on background level 100% burnt. The position of the grasshoppers in the training presentation was identical for all test subjects. All explanations and instructions to participants were identical and done by E. K. After the practice, block participants were left alone in the room for the 15 experimental presentations. The presentation program was programmed in MATLAB 2011(The MathWorks Inc, Natick, Massachusetts) and presented to the participants on a 15″ screen (Fujitsu Lifebook e series; Fujitsu limited, Tokyo, Japan).

### Human test subjects

Forty-five men (*N* = 25) and women (*N* = 20) with normal or corrected to normal vision participated in the experiment {average age 31 ± 5.3 (standard deviation [SD]) years}. Test subjects were randomly assigned to one of the five background levels, giving nine persons per level (five men and four women). There was no age difference between test persons in the different groups (*F*_4,44_ = 0.8, *P* > 0.5). We discuss the potential limitations of using humans as test subjects below (see Discussion section).

### Statistic analysis

We used survival analyses and the procedure LIFETEST in the statistical package SAS to analyze our data from the detection experiment. As indicated above, the presentation program recorded both detection time and the correctness of the answer, thus in effect images of grasshopper individuals could escape predation in two ways; if the participant made no attempt to click on the grasshopper during 60 sec or if the participant clicked somewhere on the image where there was no grasshopper. The survival analysis combined the data for time to detection and correctness of the response. Our rationale is that under natural conditions, if a predator attacks but fails to catch a grasshopper, the grasshopper can jump or fly away and avoid being eaten. An intrinsic characteristic of survival data that measure the time until an event (e.g., mortality or detection) is the possibility for censoring of observations, that is, to use those individuals even when the time until the event is not observed, for instance if the study is terminated before the prey is detected or dies (Hosmer et al. 2008). In our experiment, the subject was presented a new combination of background and grasshopper images if the grasshopper had not been detected in 60 sec. Due to the limited presentation time, the actual survival time could not be ascertained and only a lower bound on their survival time is known for these individuals. Such observations are said to be right censored (Hosmer et al. 2008). The nonparametric LIFETEST procedure takes censoring into account and uses information for both censored and uncensored observations (SAS 2004).

To determine whether the hypothesis that survival (i.e., detection time) of black grasshoppers increases when viewed against visual backgrounds that are more strongly blackened by fire should be accepted or rejected, we used the LIFETEST procedure, specified percent black in the visual background (0%, 25%, 50%, 75%, or 100%) in the TEST statement, and recorded the nonparametric Rank Tests to evaluate the association of survival time with the covariate (i.e., percent black in the background). Following the recommendation of Hosmer et al. (2008), we assessed statistical significance using the nonparametric Chi-square statistic for the log-rank test (which places more weight on differences in survival at larger values of time) and for the generalized Wilcoxon test (which places more weight on differences in survival at smaller values of time).

To facilitate comparisons and visualize the relationship linking survival of black grasshoppers to the five different backgrounds, we also carried out a stratified test, specifying percent black in the visual background in the STRATA statement, in order to obtain estimates of average survival time for each level of burnt background. However, note that mean survival times estimated using this approach are sometimes underestimated because if the largest observation is censored the estimation is restricted to the largest event time (Hosmer et al. 2008).

In our experiment, each predator contributed information from 15 sequential presentations. The results may therefore have been influenced by learning, and the repeated observations were not independent. We used two approaches to address these issues. To test and control for effects of experience, we used all observations in a single LIFETEST analyses and included sequence number as an additional covariate (besides percentage of black in the visual background) in the TEST statement. If our human predators improved their ability to detect grasshoppers as they gained experience, one would expect survival time to decrease with presentation sequence number. However, the extra variation in survival time explained when sequence number was incorporated as an additional covariate in the model was very small and far from being statistically significant both for the Log-Rank test (Chi-square increment = 0.13, *P* = 0.72) and for the Wilcoxon test (Chi-square increment = 0.0047, *P* = 0.95), indicating that effects of experience and learning were negligible. As supporting information, we show the proportion of presented grasshopper images that were detected as a function of presentation number (Fig. S1).

To ameliorate problems associated with independence of repeated observations, we performed 15 separate LIFETEST analyses and based our conclusions on the averages of these analyses, using a philosophy resembling that underlying randomization tests (Sokal and Rohlf 1981). In this approach, data for all five background levels and all 45 human predators were used for each separate analysis, but each human predator contributed only one of the 15 observations to each of the 15 analysis. We used the LIFETEST procedure with the TEST statement to compute 15 separate Rank Tests for the association of survival time with background level, one for each sequence number, and the GROUP statement to obtain 15 separate estimates of mean survival time for each of the five background levels. Next, we computed the means and the associated standard errors (SE) and interquartile ranges of the 15 separate estimated survival times for each of the five background levels. Finally, we computed the average across the 15 separate rank test statistics (i.e., Chi-square values for the Wilcoxon and Log-Rank tests) and assessed the associated statistical significance against one degree of freedom (Hosmer et al. 2008).

In the analytical approach outlined above, estimated survival time may have increased if time to detection increased or if an increased proportion of prey escaped detection altogether. To evaluate the hypothesis that time to detection for those prey that were actually detected increased with increasing degree of burnt substrate in the visual background images, we used regression analyses, and included in the model both linear and nonlinear (quadratic) effects. In this approach, each human predator contributed one observation (the average time to detection across all [maximum 15] detected prey images) to the analyses. Data for censored observations (i.e., when prey were not detected within the 60-sec presentation time) were excluded from this analysis. We used Spearman's rank correlation test to evaluate the hypothesis that the percent of undetected grasshopper images increases with increased degree of burnt substrates in the background images. Additionally, we used regression analyses to examine if there was any nonlinear quadratic effect of black substrate in the background on percentage undetected prey. Each human predator contributed one observation (the percent undetected prey images of 15 presentations) to this analysis.

## Results

Our results are based on data for a total of 675 interactions between human predators and images of black grasshoppers presented in random positions on images of natural backgrounds representing five different degrees of recovery after fire. On average, 55% of the presented grasshopper images were detected (“eaten”) by the human predators. Overall, we found differences in estimated survival, time to detection, and percent undetected images of grasshoppers among the different backgrounds.

**Figure 2:**
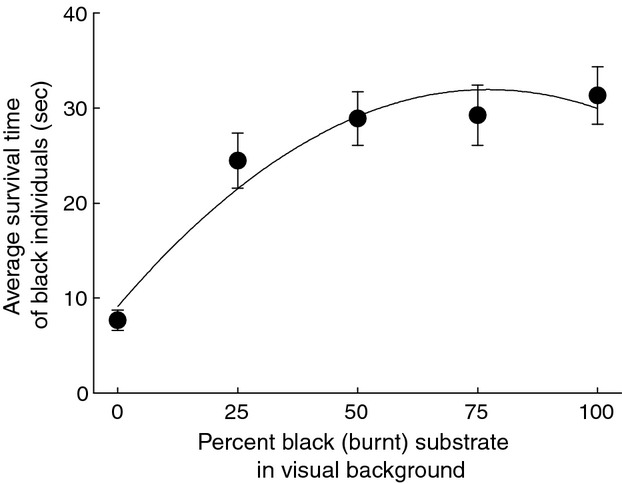
Estimated survival times for images of black *Tetrix subulata* pygmy grasshoppers presented on a computer screen to human predators against images of natural backgrounds containing substrate that had been blackened by fire to different degrees (0%, 25%, 50%, 75%, or 100%). Figure shows mean (±SE) of 15 separate estimates of average survival time for each of the five background levels. The curved line denotes the fitted second order regression line. Prey images were presented for a maximum of 60 sec and survival times were estimated using LIFETEST analysis in SAS. Note that mean survival times are sometimes underestimated because if the largest observation is censored the estimation is restricted to the largest event time. See text for details.

As expected, if black coloration provides better protection against visually oriented predators in environments where the surface substrate has been blackened by fire, estimated survival times of black grasshoppers presented on images of natural backgrounds increased continuously with increasing levels of black substrate in the backgrounds (LIFETEST, Rank Tests for the association of survival time with degree of black in background, reported test statistics are averages based on the results of separate analyses for each of 15 presentation sequences: Log-Rank test, *X*^2^ = 8.59, degrees of freedom (df) = 1, *P* < 0.005; Wilcoxon, *X*^2^ = 9.88, df = 1, *P* < 0.005, the Wilcoxon test was statistically significant in 13 of the 15 separate tests, median *P* = 0.0098, *P* range < 0.0001–0.58) (Fig. 2). Regression analyses based on the estimates of mean survival times uncovered statistically significant linear (*F*_1,72_ = 24.18, *P* < 0.0001) and nonlinear (*F*_1,72_ = 11.78, *P* = 0.001) effects of black substrate in the background on survival time (y = 9.6 + 0.61x−0.004x^2^), and the proportion of the total variance explained by the model (as estimated by *R*-squared) increased from 0.28 to 0.38 when the quadratic factor was included.

**Figure 3:**
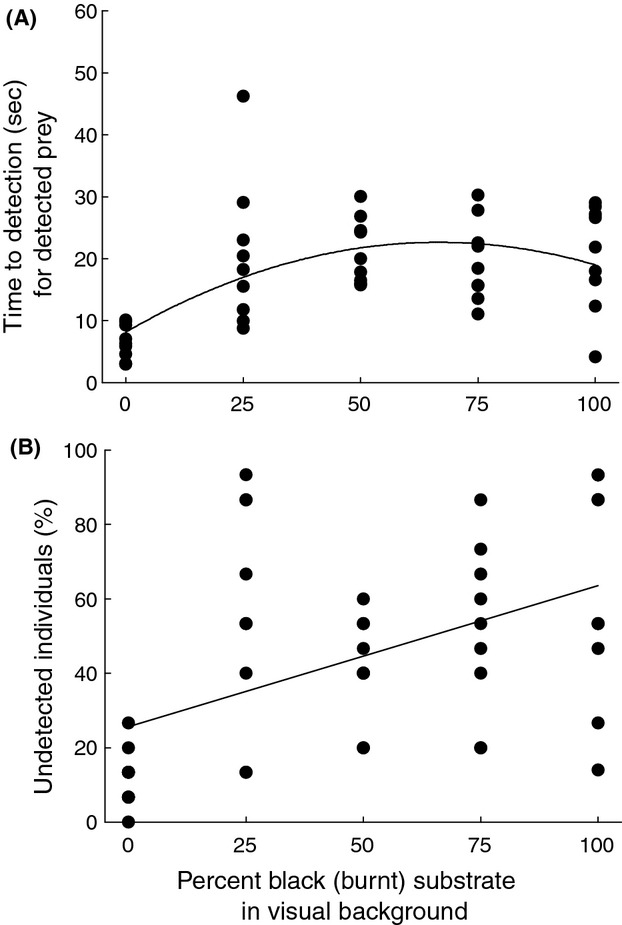
Human predators needed more time to detect the black *Tetrix subulata* pygmy grasshopper images and more frequently failed to detect the grasshopper images when they were presented against computer images of backgrounds that were more heavily blackened by fire. (A) Average time to detection increased nonlinearly with increasing degree of black substrate in the backgrounds. Each symbol represents the mean value for a single human predator presented with 15 images. The curved line denotes the fitted second order regression line. (B) The percentage of black grasshoppers that were not detected by human predators increased when presented against blacker backgrounds. Each symbol represents the percentage of undetected grasshoppers of 15 presentations for each human predator. See text for details.

Survival time increased with increasing degree of black substrate in the visual backgrounds because the human predators both needed more time to detect the grasshopper and more frequently failed to detect the grasshopper when it was presented against images of backgrounds that were more heavily blackened by fire (Fig. 3). The average time to detection for those prey that were detected (i.e., excluding data for censored observations) increased asymptotically to about 20 sec with increasing degree of burnt substrate in the visual background images (Fig. 3A). Regression analyses uncovered statistically significant linear (*F*_1,42_ = 14.39, *P* = 0.0005) and quadratic (*F*_1,42_ = 8.82, *P* = 0.005) effects of black substrate in the background on detection time (y = 8.2 + 0.44x−0.003x^2^), and the proportion of the total variance explained by the model (as estimated by *R*-squared) increased from 0.18 to 0.32 when the quadratic factor was included. The average percent of presented grasshopper images that remained undetected by human predators increased with increasing degree of burnt substrate in the background images (*r*_s_ = 0.50, *P* = 0.004, *N* = 45) (Fig. 3B). Results from regression analyses further indicate that while there was a significant linear effect (*F*_1,42_ = 5.89, *P* = 0.02), there was no statistically significant quadratic effect of black substrate in the background on percentage undetected prey (*F*_1,42_ = 2.09, *P* = 0.16).

## Discussion

In this study, we presented human “predators” with images of black *T. subulata* pygmy grasshoppers against samples of their natural habitats on computer screens. Our results demonstrate that risk of being detected depends on the visual background, and shows that survival of melanistic individuals increase asymptotically with increasing degree of burnt substrate in the environment. The human predators both needed more time to detect the grasshopper and more frequently failed to detect the melanistic grasshoppers presented against backgrounds that were more heavily blackened by fire. This corroborates the notion that visual predators may impose divergent and oscillating selection on color pattern due to environmental changes stemming from fire events, and that camouflage may drive the evolutionary shifts in melanism frequencies that have been documented in replicated populations of *T. subulata* pygmy grasshoppers (Forsman et al. 2011).

We have demonstrated that against a nonburnt greenish background, the black grasshoppers are extremely easy to detect. The variation between different predators in the ability to detect black grasshoppers on the nonburnt backgrounds was very low, relative to the other background conditions. Furthermore, it seems that even a relatively small proportion (e.g., 25%) of blackened surface area is sufficient to impair the ability of predators to detect black grasshoppers. This suggests that it may not be necessary for a melanistic grasshopper to blend into its immediate background to escape predation, but it may also benefit from the presence of dark patches and small, sooty burnt twigs, and needles as these may impair prey recognition. It is likely that some of the “wrong” answers given by our test subjects before the time limit had been reached represented misidentifications, as has been suggested in other cases of masquerade (e.g., Stevens and Merilaita 2009; Skelhorn et al. 2010a,b). We propose that both background matching and masquerade may contribute to reduced predation and increased survival of black grasshoppers in burnt and partly burnt (black) environments.

To our knowledge, this study is one of the first attempts to experimentally investigate changes in crypsis and predation risk associated with changing visual environments resulting from ecological succession. The rise and fall of the melanistic morph of the Peppered Moth *B. betularia* associated with atmospheric pollution and lichen succession in the UK and USA (Kettlewell 1973; Grant et al. 1995; Majerus 1998; Grant and Wiseman 2002), offers another possible example of such oscillating selection. Thus, melanistic moths became common during the industrial revolution when environments became sooty due to air pollution, and experimental studies indicate that during the 1950s differential predation favored melanistic moths (Kettlewell 1955, 1956). Since 1979 there has been a decline in the frequency of the melanistic form, and accordingly, results of a more recent experiment conducted by Michael Majerus during 2001–2007 indicate that there is now strong differential bird predation against melanistic peppered moths (Cook et al. 2012). Although there are similarities between industrial melanism in moths and fire melanism in grasshoppers, the change in direction of selection and the evolutionary shifts in melanism frequencies are replicated in several independent populations and occur at a much faster rate in pygmy grasshoppers (Forsman et al. 2011).

### Potential limitations of using humans as predators

To use humans as “predators” in image-based detection experiments has proven to be an effective tool in studies of protective coloration (e.g., Knill and Allen 1995; Beatty et al. 2005; Bohlin et al. 2008; Todd 2009; Tsurui et al. 2010; Bohlin et al. 2012). However, the approach lacks in realism, and will generate useful results and valid conclusions only under the assumptions that differences in rates of detection of (motionless) prey images by humans are correlated with rates of detection, and capture, by natural predators of live prey animals in the wild. Their small size and locally high population densities render grasshoppers susceptible to visual predators, such as birds (Isley 1938; Bock et al. 1992) and lizards (Civantos et al. 2004). Invertebrate predators such as spiders may also be an important source of grasshopper mortality (Belowsky and Slade 1993), but they probably do not select their prey on the basis of color pattern. In contrast with humans, some insectivorous predators, such as birds, are sensitive to ultraviolet light (Bennett and Cuthill 1994). However, Tsurui et al. (2010) measured very small reflectance of ultraviolet from Tetrix grasshoppers, and it is therefore reasonable to assume that there exists an overlap between how humans and natural predators perceive their color patterns. Humans and natural predators also share similar information processing capabilities (Dukas and Kamil 2001). That results from previous research in which humans were used as predators (Knill and Allen 1995; Beatty et al. 2005) did not deviate substantially from the findings of similar studies using birds (Bond 1983; Alatalo and Mappes 1996) further support the notion that humans may be successfully used as substitutes for natural predators. However, given the increasing popularity of the approach, a systematic evaluation of its underlying assumptions is called for.

## Conclusions

In conclusion, our findings implicate camouflage and predation as an important driver of fire melanism in pygmy grasshoppers. Using humans as “predators” on images of prey items presented on samples of natural background on computer screens offers a powerful method to test hypotheses about predation and selection that are difficult or impossible to investigate with sufficient level of replication under natural conditions in the wild. Our present results add to the existing body of evidence that the color pattern polymorphism and incidence of melanistic pygmy grasshoppers is under the influence of selection imposed by visually oriented predators (Forsman and Appelqvist 1998, 1999; Civantos et al. 2004; Tsurui et al. 2010). Our findings are highly consistent with the recent study by Forsman et al. (2011), who reported higher frequencies of black individuals in *T. subulata* populations in recently burnt than in nonburnt habitats. That populations in nonburnt, greenish areas had very low frequencies of black grasshoppers can be explained by the ease with which they are detected or recognized in such environments. Our demonstration that predation risk of black grasshoppers declined with decreasing proportion burnt substrate in the background can also account for the gradual decline in the frequency of black grasshoppers recorded in natural populations several years after fires (Forsman et al. 2011). Our results highlight the continued value of animal color patterns, especially in respect to melanism, in enhancing our understanding of the role of selection in changing environments for the evolution of adaptations and maintenance of biological diversity.

## Supporting Information

**Figure S1.** Proportion of presented grasshopper images that were detected as a function of presentation number.
