# Supplementary material for: Reduced predation risk for melanistic pygmy grasshoppers in post-fire environments
Source: Ecol Evol. 2012 Aug 1;2(9):2204–12. doi: 10.1002/ece3.338 (PMC3488671; doi:10.1002/ece3.338)
Supplement: Supplementary file 1 [file ece30002-2204-SD1.pdf]

Supporting Information – Figure S1

REDUCED PREDATION RISK FOR MELANISTIC PYGMY GRASSHOPPERS IN POST-FIRE ENVIRONMENTS

Einat Karpestam [1], Sami Merilaita [2] and Anders Forsman [1,3]

[1] *School of Natural Sciences, Linnaues University, SE-391 82 Kalmar, Sweden*

[2] *Environmental and Marine Biology, Åbo Akademi University, FI-20520 Turku, Finland*

[3] *Corresponding author E-mail: Anders.Forsman@lnu.se*

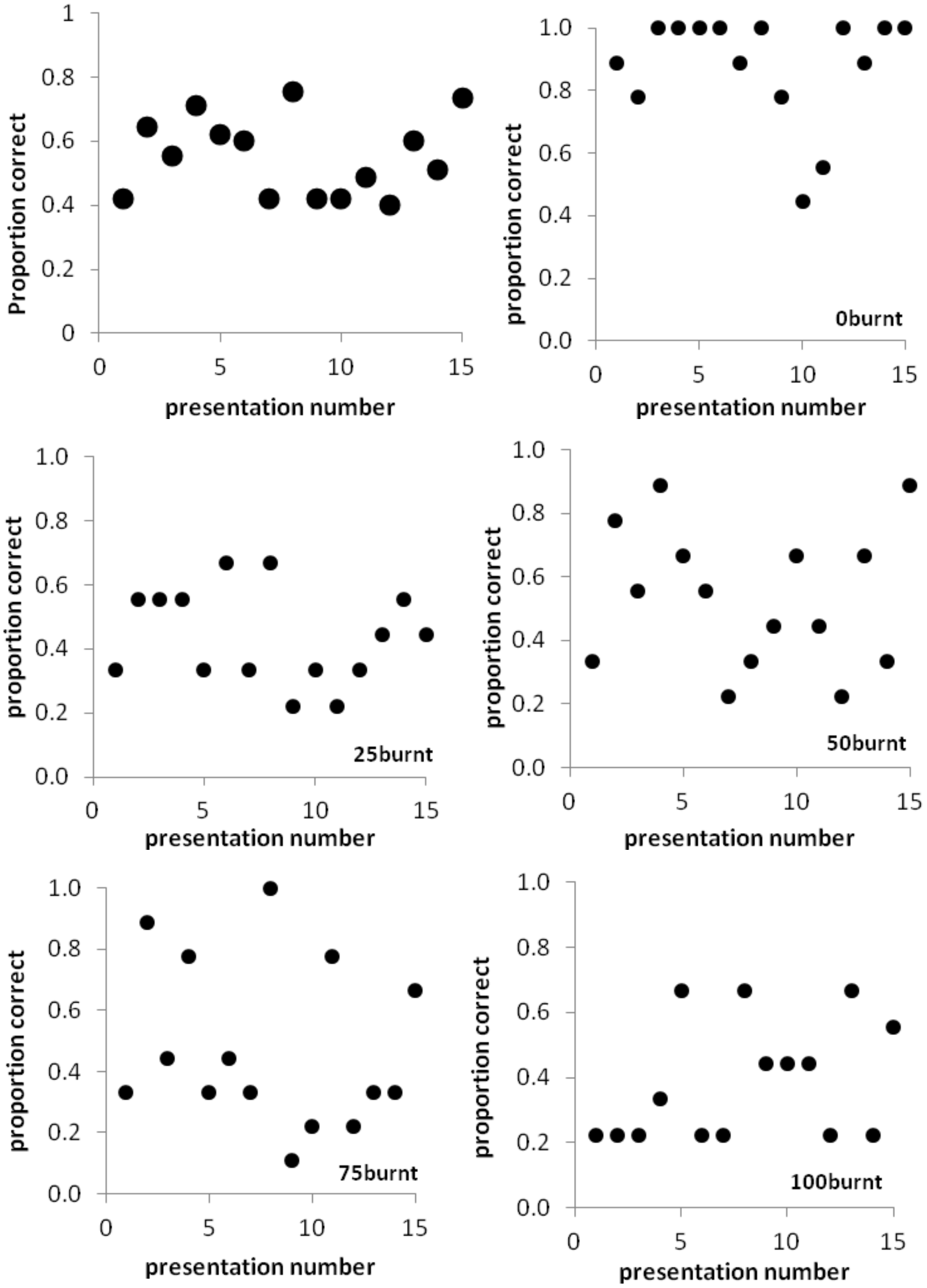

Figure S1. Proportion of detected grasshopper images as a function of presentation number. Mean proportion of presented grasshopper images that were detected computed across human predator individuals and backgrounds as a function of presentation number (A). Mean proportion of presented grasshopper images that were detected as function of presentation number across individuals on each separate background (0, 25, 50, 75, or 100% burnt substrate).
