# Supplementary figures and images for: Reduced predation risk for melanistic pygmy grasshoppers in post-fire environments
Source: Ecol Evol. 2012 Aug 1;2(9):2204–12. doi: 10.1002/ece3.338 (PMC3488671; doi:10.1002/ece3.338)

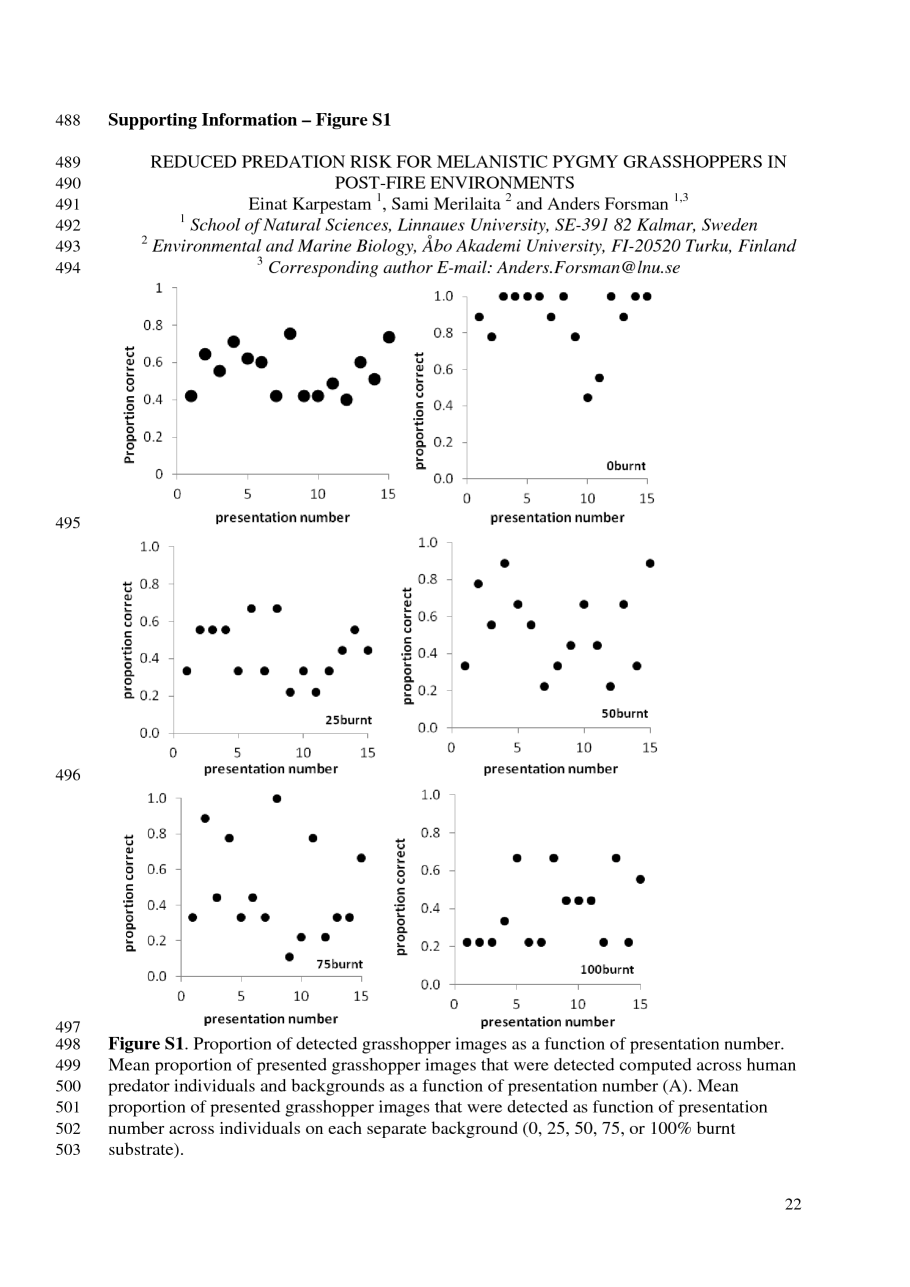

Supplement: Supplementary file 2 [file ece30002-2204-SD2.png]
